# Investigation of 3-year inpatient TB cases in Zunyi, China: Increased TB burden but improved bacteriological diagnosis

**DOI:** 10.3389/fpubh.2022.941183

**Published:** 2022-08-02

**Authors:** Ling Chen, Xiaodan Wang, Xudong Jia, Yuanbo Lan, Haibo Yi, Xiaomin Wang, Peng Xu

**Affiliations:** ^1^Tuberculosis Division of Respiratory and Critical Care Medicine, Affiliated Hospital of Zunyi Medical University, Zunyi, China; ^2^School of Basic Medicine, Zunyi Medical University, Zunyi, China

**Keywords:** tuberculosis (TB), tendency, rifampicin-resistant TB, epidemic, diagnosis

## Abstract

**Background:**

As one of the top three high tuberculosis (TB) burden countries, China is a country where the overall TB incidence continues to decline. However, due to its large population and area, the increased TB burden exists in regional areas.

**Methods:**

This retrospective study analyzed local inpatient pulmonary TB cases in the Affiliated Hospital of Zunyi Medical University (AHZMU) from January 2016 to December 2018 in a high TB incidence and economically-less-developed area of China. Four methods, acid-fast bacilli stain, culture, Xpert and LAMP, were used to detect *Mycobacterium tuberculosis* (*M.tb*), while proportional method and Xpert were used to identify rifampicin-resistant TB (RR-TB). Case number, treatment history, *M.tb* confirmed TB and rifampicin resistant proportion were analyzed to investigate the local TB epidemic.

**Results:**

Total 3,910 local inpatient cases with pulmonary TB were admitted to AHZMU during this study period. The annual numbers of total TB cases increased 26.4% (from 1,173 to 1,483), while new cases increased 29.6% (from 936 to 1,213) and RR-TB cases increased 2.7 times (from 31 to 84). Meanwhile, the percentage of previously treated cases declined from 20.2 to 18.2% and the *M.tb* confirmed TB proportion increased from 34.7 to 49.7%.

**Conclusion:**

The elevated *M.tb* confirmed TB proportion and the declined percentage of previously treated cases indicated the improved TB diagnosis and treatment of AHZMU. However, the increasing number of total TB cases, new and RR-TB cases showed an upward trend and increased TB burden in a relatively underdeveloped area of China.

## Introduction

Tuberculosis (TB), caused by *Mycobacterium tuberculosis* (*M.tb*), has been a serious threat to human health since ancient times. In the past decade, more than 10 million people have died from this disease worldwide. To end the global TB epidemic, the End TB strategy was mapped with the aim of reducing the global incidence by 90% by 2035 compared to the 2015 baseline. However, there were still 10 million people who developed TB disease in 2019 (1), besides that, the COVID-19 pandemic has impacted progress toward achieving the goals set in the END TB strategy.

Although China is one of the top three high TB burden countries, the TB incidence had continually declined from the year 2000 to 2019 (1), making important efforts to global TB control. Due to the diversity of economics, culture and geography in China, the regional variances in TB epidemic status also exist. Guizhou Province, located in Southwest China, an economically-less-developed area, is one of the regions with the most severe TB epidemic (2). The incidence rate of Guizhou Province is 134/100,000 population, ranking third in China, more than two times higher than the national average (63/100,000 population) (3). Zunyi City, the second largest city of Guizhou Province. This retrospective study analyzed the 3-years data of the local (Zunyi City) TB inpatients of the Affiliated Hospital of Zunyi Medical University (AHZMU), to investigate the current TB regional epidemic situation and risk factors for rifampicin (RIF) resistance. As a key designated hospital for TB diagnosis and treatment in Guizhou Province, the AHZMU is the main TB hospital in Zunyi. Therefore, the data of AHZMU can be an indicator of the local TB epidemic.

## Materials and methods

### Data collection

From January 1, 2016 to December 31, 2018, a total of 3,910 inpatient cases from Zunyi were diagnosed as pulmonary TB in AHZMU. Demographic and clinical data of patients were collected. The data collection and procedures were described previously (4). Any information related to patient identity was filtered to ensure the data used in this study were anonymous. In order to avoid duplicated cases, each patient was assigned a unique hospital admission number, which was used to organize all patient data. This study has been approved by the ethics committee of Zunyi Medical University.

### Laboratory procedure

For TB or presumptive TB patients, sputum samples were collected for *M.tb* detection. The specimen procedure was performed according to the previous study (5). Bacteriological testing methods included acid-fast bacilli (AFB) stain, Löwenstein-Jensen (L-J) solid media culture, GeneXpert MTB/RIF (Xpert) (Cepheid Inc. USA) and Loopamp MTBC Detection Kit (LAMP) (Eiken Chemical Co. Japan). AFB stain and culture followed the WHO recommended procedures, while Xpert and LAMP were operated according to the instruction manual. To distinguish *M.tb* and non-tuberculosis mycobacteria (NTM), 2-thiophene carboxylic acid (TCH) and the p-nitrobenzoic acid (PNB) selective L-J media was used among cultural positive cases. And cases with identical sample that was AFB positive but molecular testing (Xpert or LAMP) negative, were not identified as *M.tb*. Cases with doubtful results required additional tests.

The RIF susceptibility testing of *M.tb* was conducted by traditional culture-based method and/or Xpert. Strictly according to the WHO recommended method, culture-based RIF susceptibility testing was proportional method, performed on L-J solid medium with a RIF concentration of 40 μg/ml. Cases with either phenotypic DST or Xpert of RIF resistant were identified as RIF resistance.

### Clinical diagnosis of pulmonary TB

The clinical diagnostic criteria of pulmonary TB were judged by clinical features, imaging results and laboratory tests, which were according to the health industry standards of China (WS 288-2008 and WS 288-2017). NTM disease was excluded in this study which was diagnosed by bacteriological diagnosis and/or clinical evidence.

### Literature review of rifampicin-resistant TB trends in China

To compare our result of RR-TB trend in other regions of China during the similar period, we reviewed articles published in recent years. We searched for eligible studies from PubMed and two Chinese Literature Databases (CNKI, https://www.cnki.net/; WANFANG, https://www.wanfangdata.com.cn/) by the following search terms: (“tuberculosis” or “TB”) AND (“rifampicin” or “RIF”) AND (“China” or “Chinese”). Studies targeting special populations, such as children or HIV-infected patients, were excluded. Only studies with multiple data of RR-TB proportions among years were included. The proportions of RR-TB at the beginning and end of each study were collected.

### Statistics

Stata (version 16.0, StataCorp. USA) was used for statistical analysis. The trends of new and previously treated cases, *M.tb* positive and negative cases, rifampicin-susceptible and -resistant TB cases were analyzed by chi-square test. Since RIF susceptibility is important in treatment outcomes and TB control, multiple statistical approaches (6), logistic, Poisson, modified Poisson, and log-binomial regression, were applied to analysis of the characteristics associated with RIF resistance. The univariate and multivariate regression were used to calculate odds ratio (OR), adjusted odds ratio (AOR), prevalence ratio (PR), adjusted prevalence ratio (APR), and 95% confidence interval (CI) for factors associated with RR-TB.

## Results

**Figure 1:**
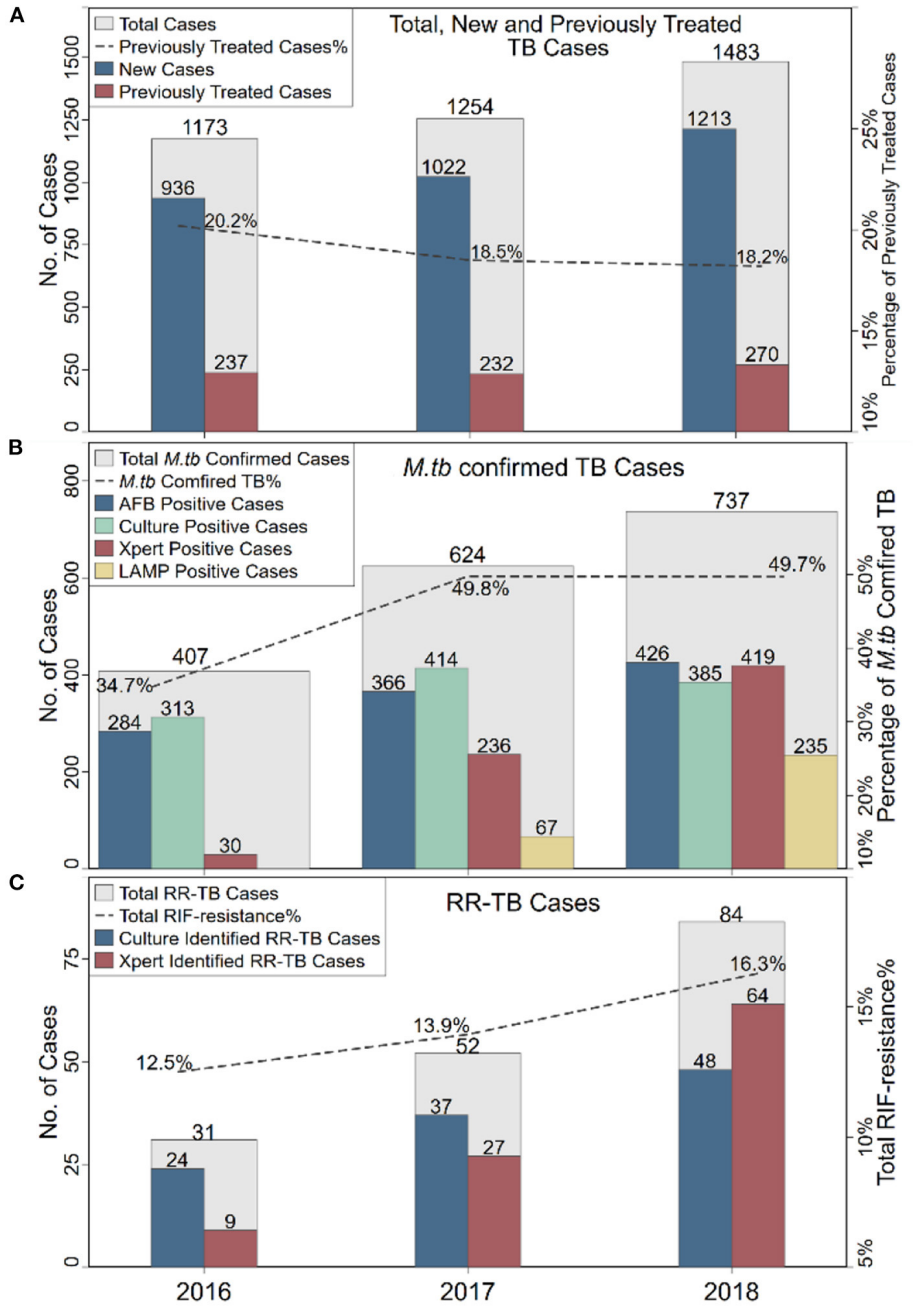
The annual distributions of TB cases **(A)**, *M.tb* confirmed TB cases **(B)** and RR-TB cases **(C)**. TB, tuberculosis; RR-TB, rifampicin-resistant TB; AFB, acid-fast bacilli.

From 2016 to 2018, 3,910 inpatient cases from Zunyi were diagnosed as pulmonary TB in AHZMU. To investigate the local TB tendency, the annual distribution of TB cases was analyzed. The total number of TB inpatients increased 26.4%, from 1,173 cases in 2016 to 1,483 in 2018 (Figure 1A). The new TB cases increased 29.6%, from 936 to 1,213, which was a similar pattern to the overall TB cases. In contrast, the number of previously treated cases was relatively stable, and its percentage declined from 20.2 to 18.2%. No statistical significance (*P* = 0.2026) was revealed between new and previously treated cases among years (Supplementary Table 1).

**Figure 2:**
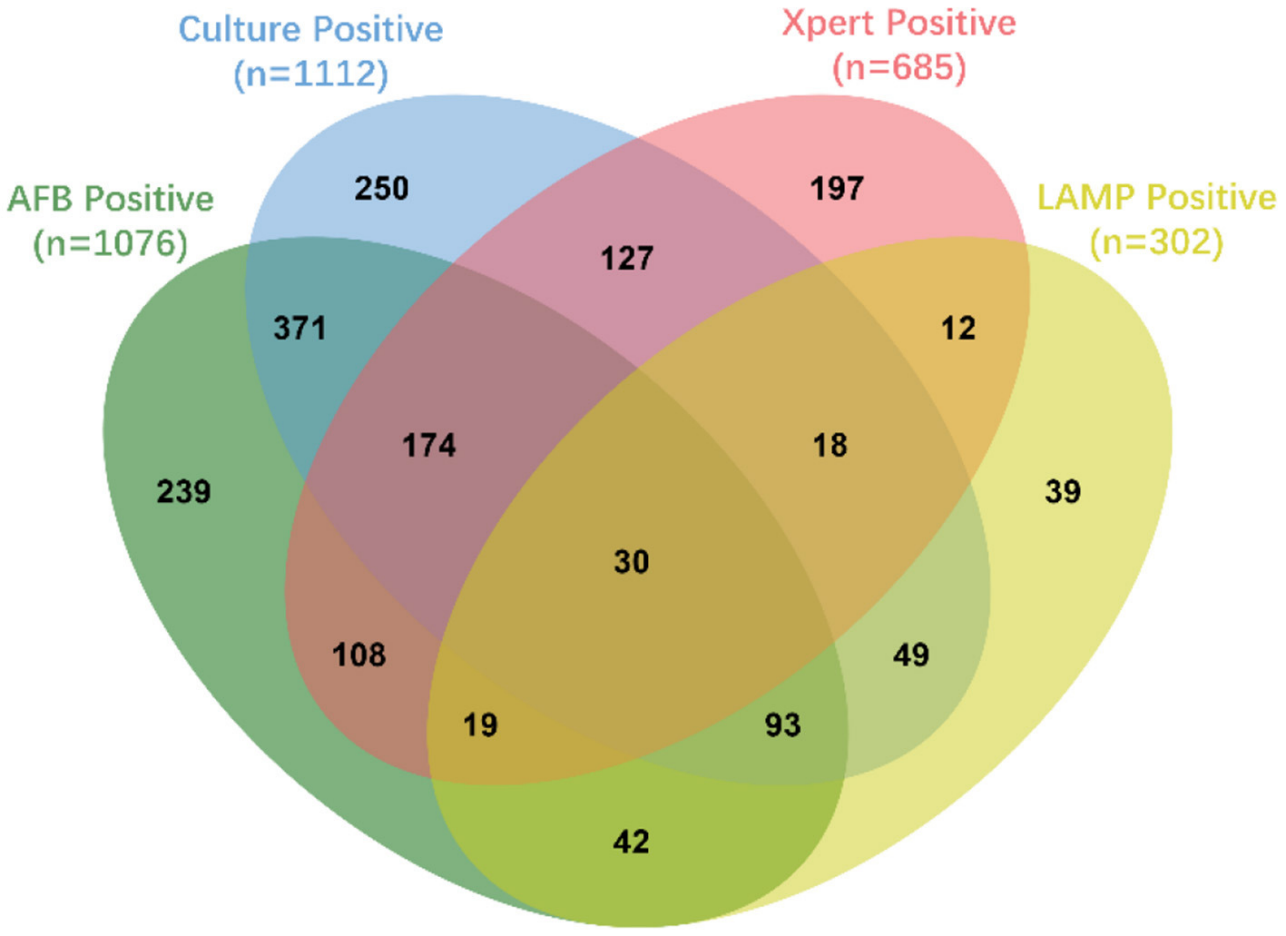
The Venn diagram of *M.tb* positive TB cases. This Venn diagram was created by jvenn web application (7). The oval-shaped areas and numbers indicated the *M.tb* positive TB cases that were detected by different methods, which green indicated acid-fast bacilli (AFB), blue indicated Löwenstein-Jensen (L-J) solid media culture, pink indicated GeneXpert MTB/RIF (Xpert) and yellow indicated Loopamp MTBC Detection Kit (LAMP).

Pathogen detection is an important criterion for TB diagnosis. In this investigation, four methods were used to detect *M.tb*. There was AFB stain, L-J solid media culture, Xpert and LAMP (since 2017). Almost every case (3,841/3,910, 98.2%) applied at least one of these four methods. A total of 1,768 cases were *M.tb* positive. More than half (1,043/1,768, 59.0%) of *M.tb* positive cases were verified by two or more methods (Figure 2). The overall *M.tb* confirmed TB proportion was 45.2% (1,768/3,910), which was increased from 34.7% in 2016 to 49.7% in 2018 (Figure 1B). There was a significant difference between *M.tb* positive and negative cases (*P* < 0.0001) among years (Supplementary Table 1).

**Figure 3:**
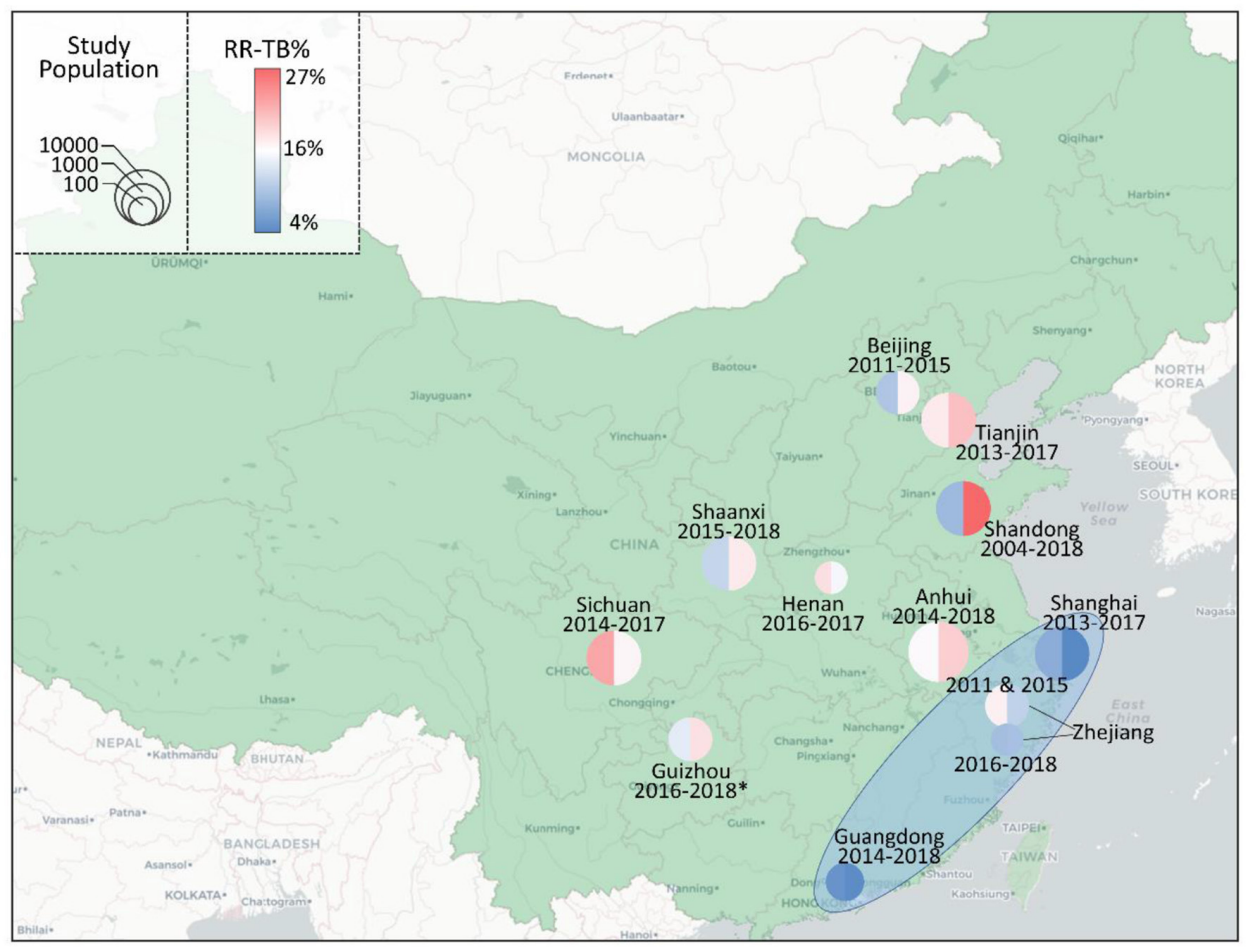
The proportions of RR-TB in different areas of China. The proportions of RR-TB at the beginning (left) and end (right) of each study were marked on the map of China by the color of the circle. A warmer color indicated a higher RR-TB proportion, while a cooler color indicated the opposite. The size of cycles indicated the size of the study population. The studied periods and the provinces were labeled next to the circles. The light blue oval area indicated the southeastern coast of China, where studies showed decreased or stable RR-TB proportions. The data of Anhui was obtained from Meng (8), Beijing from Zhang et al. (9), Guangdong from Han et al. (10), Guizhou from this study (*), Henan from Wang et al. (11), Shaanxi from Lei et al. (12), Shandong from Song et al. (13), Shanghai from Wang et al. (14), Sichuan from Zhou et al. (15), Tianjin from Bai et al. (16), Zhejiang (2011 and 2015) from Li et al. (17) and Zhejiang (2016–2018) from Zheng et al. (18).

In addition to the number of TB cases, the proportion of drug-resistant TB, especially the RR-TB, is also a key indicator of the TB burden. In this retrospective study, culture-based phenotypic drug-susceptibility test (DST) and/or molecular DST by Xpert were used to test RIF susceptibility. Among 1,112 *M.tb* culture positive cases, 60.1% (668/1,112) were subjected to phenotypic DST, of which 16.3% (109/668) were RR-TB. Among 685 Xpert *M.tb* positive cases, 14.6% (100/685) were RIF resistant. In total, 167 cases were RR-TB, of which 42 cases were identified by both phenotypic DST and Xpert. The number of RR-TB cases increased dramatically, which increased 2.71 times, from 31 in 2016 to 84 in 2018. Among all RIF susceptibility tested cases, the total RR-TB proportion was 14.7% (167/1,137), which increased from 12.5% in 2016 to 16.3% in 2018 (Figure 1C). However, no statistical significance (*P* = 0.1456) was revealed between rifampicin-susceptible and -resistant TB cases among years (Supplementary Table 1). To figure out whether this phenomenon of increased RR-TB proportion was unique in China. A total of 11 articles published in recent years with RR-TB proportions among years were found (8–18). There was a regional variation of RR-TB proportions in China (Figure 3). Interestingly, all four studies from the southeastern coast of China (one of the most developed areas of China) showed decreased or stable RR-TB proportions. On the contrary, among the rest studies (including this study), 75% showed upward trends in recent years. In order to identify the factors related to RIF resistance in this study, univariate and multivariate logistic regression were analyzed (Table 1). Previously treated patients had the highest odds (AOR 4.24, *P* < 0.0001) of being RR-TB compared to new patients, while patients over 60 years had a lower likelihood (AOR 0.43, *P* = 0.012) compared to those 20 years of age. Poisson, modified Poisson, and log-binomial regression also identified the same factors related to RIF resistance with minor differences in PR, Adjust PR and *P*-Value (Supplementary Table 2).

**Table 1:** Univariate and multivariate logistic regression analysis of characteristics associated with rifampicin resistance.

**Characteristic**	**RIF susceptible**		**RIF resistant**		**Total**	**OR (95% CI)**	***P-*Value**	**Adjust OR (95% CI)**	***P*-Value^a^**
	**No**.	**%**	**No**.	**%**					
**Gender**									
Male	612	85.2%	106	14.8%	718	1.00		1.00	
Female	358	85.4%	61	14.6%	419	0.98 (0.70–1.38)	0.925	1.04 (0.72–1.49)	0.829
**Age**									
≤ 20	108	86.4%	17	13.6%	125	1.00		1.00	
21–40	237	78.5%	65	21.5%	302	1.74 (0.98–3.11)	0.061	1.49 (0.82–2.72)	0.192
41–60	284	83.0%	58	17.0%	342	1.30 (0.72–2.33)	0.382	0.93 (0.50–1.72)	0.816
≥61	341	92.7%	27	7.3%	368	0.50 (0.26–0.96)	**0.037**	0.43 (0.22–0.83)	**0.012**
**Treatment history**									
New case	786	90.3%	84	9.7%	870	1.00		1.00	
Previously treated case	184	68.9%	83	31.1%	267	4.22 (2.99–5.95)	**<0.0001**	4.24 (2.97–6.05)	**<0.0001**
**Total**	970	85.3%	167	14.7%	1,137				

^a^P-values in bold denote statistical significance at the P < 0.05 level. RIF, rifampicin; CI, confidence interval; OR, odds ratio.

## Discussion

As an important feature of TB epidemic status, the total number of pulmonary TB cases showed an upward trend in this study. Briefly, high TB transmission leads to more new TB cases, while poor treatment results in more previously treated cases. By analyzing the treatment history, we found that the increase in TB cases was mainly caused by new TB cases. In addition, both the number and proportion of RR-TB cases also increased. Unfortunately, this phenomenon of increased RR-TB proportion was not unique in China. Through literature review, we found that the increased RR-TB proportion was common in the middle and west of China. Our findings not only revealed the tendency of local TB epidemic in a relatively underdeveloped area of China, but also indicated that even in countries with continuously declined TB incidence rate, there is still a risk that the trends of TB epidemic will turn upward in some areas.

Nevertheless, there is some good news. During the 3-year-studying period, the *M.tb* confirmed TB proportion was elevated from 34.7 to 49.7%, and the percentage of previously treated cases declined from 20.2 to 18.2%, which indicated improvement in bacteriological TB diagnosis and treatment of AHZMU. These improvements were potentially due to the application of molecular methods (Xpert and LAMP) and the accumulated clinical experience, which would have positive effects on local TB control. Nevertheless, hospitals are only one part of the TB control system. To control the local TB epidemic more effectively, more efforts need to be exerted, such as reducing the number of undetected TB patients and the risk of transmission, screening high-risk population, and monitoring close contacts.

Globally, the incidence rate of TB was reducing in both the high and low TB burden countries, such as China, India, and the United States (19). According to WHO's report, the TB incidence in China has dropped from 75/100,000 in 2011 (20) to 58/100,000 in 2019 (1). A similar decline has also been observed in many provinces and cities of China (21–24). However, this is not a universal phenomenon. In western China, one of the regions with the highest TB epidemic, Yang et al. (25) found an increased TB incidence in many areas from 8-year-data. In southwest China, our data also showed an upward trend of TB burden in Zunyi. These results indicated that in some regions of China, particularly the regions with high TB epidemic, the trends of TB epidemic might turn upward, which would be an obstruction to the national TB control and need to be paid more attention to.

RR-TB can cause serious consequences such as treatment failure, prolonged course of treatment, and high risk of relapse (26). Disturbingly, our data showed that the number of RR-TB in Zunyi rose from 31 to 84 in only three years of time. Although additional RR-TB cases were identified by Xpert, the number of RR-TB cases detected by phenotypic DST increased during the study period, suggesting that this increase in RR-TB was not only due to the application of the new detection method, but also to the presence of more RR-TB cases. In addition to the cases number, the percentage of RR-TB increased from 12.5 to 16.3%. This proportion would be a better indicator of RR-TB burden, which could reduce the disruption from additional methods or increased number of tested cases. Unfortunately, through literature review, we found that there was a general increase in RR-TB proportion in most regions except for the southeastern coast of China. Since the southeastern coast of China is one of the most developed regions in China, economic factors may be one of the reasons for the regional differences in RR-TB proportions. To treat RR-TB patients, more expensive anti-TB drugs and prolonged treatment are needed, which requires more public resources and places a heavier financial burden on patients.

One limitation of this investigation is that data was collected and analyzed only from hospitalized TB patients. Compared to outpatients, inpatient TB cases are under more serious TB conditions. It is possible that the proportion of inpatients with RR-TB is higher than the regional average. Nevertheless, the aim of this study was mainly to figure out the local epidemiological trends of TB in an underdeveloped region of China. As a 3,500-bed tertiary general hospital, AHZMU is one of the major medical centers of the healthcare system in Guizhou Province. Thus, the number of TB cases per year can be used as an indicator of TB burden. In addition, this hospital, designated by the provincial government, has been a main part of the regional referral system, specializing in the diagnosis and treatment of TB, particularly multidrug-resistant (resistance to both rifampicin and isoniazid) TB (MDR-TB) patients. Most patients with RR-TB require at least 1 month of inpatient treatment at AHZMU. Therefore, the increase in the proportion of RR-TB in this study could be an indicator of the rising trend of TB burden in Zunyi city.

## Data availability statement

The original contributions presented in the study are included in the article/Supplementary material, further inquiries can be directed to the corresponding authors.

## Author contributions

LC, XiaomW, and PX conceived and designed the study, contributed substantially to the preparation of tables and figures, and the critical revision of the manuscript. LC and PX wrote the draft manuscript. All authors were involved in collection and analysis or interpretation of the data.

## Funding

This work was supported by grants from Guizhou Provincial Science and Technology Foundation (Grant Number Qiankehe jichu [2019]1465), National Nature Science Foundation of China (Grant Numbers 31700130, 81760003, 31860706, and 81860004), Guizhou Provincial Education Department (Grant Numbers Qianjiaohe KY[2017]190 and KY[2018]222), and Science and Technology Fund Project of Guizhou Provincial Health Commission (Grant Number gzwjkj[2019]-1-042 and gzwkj[2022]- 260). The funders of the study had no role in study design, data collection, data analysis, data interpretation or writing of the report.

## Conflict of interest

The authors declare that the research was conducted in the absence of any commercial or financial relationships that could be construed as a potential conflict of interest.

## Publisher's note

All claims expressed in this article are solely those of the authors and do not necessarily represent those of their affiliated organizations, or those of the publisher, the editors and the reviewers. Any product that may be evaluated in this article, or claim that may be made by its manufacturer, is not guaranteed or endorsed by the publisher.
